# Anticancer Indole-Based Chalcones: A Structural and Theoretical Analysis

**DOI:** 10.3390/molecules24203728

**Published:** 2019-10-16

**Authors:** Farid A. Badria, Saied M. Soliman, Saleh Atef, Mohammad Shahidul Islam, Abdullah Mohammed Al-Majid, Necmi Dege, Hazem A. Ghabbour, M. Ali, Fardous F. El-Senduny, Assem Barakat

**Affiliations:** 1 Department of Pharmacognosy, Faculty of Pharmacy, Mansoura University, Mansoura 35516, Egypt; faridbadria@gmail.com; 2 Department of Chemistry, Faculty of Science, Alexandria University, P.O. Box 426, Ibrahimia, Alexandria 21321, Egypt; saied1soliman@yahoo.com; 3 Department of Chemistry, College of Science & Arts, King Abdulaziz University, P.O. Box 344, Rabigh 21911, Saudi Arabia; 4 Department of Chemistry, College of Science, King Saud University, P.O. Box, 2455, Riyadh 11451, Saudi Arabia; aboatef2008@gmail.com (S.A.); mislam@ksu.edu.sa (M.S.I.); amajid@ksu.edu.sa (A.M.A.-M.); maly.c@ksu.edu.sa (M.A.); 5 Department of Physics, Faculty of Arts and Sciences, Ondokuz Mayıs University, Atakum, Samsun 55139, Turkey; necmid@omu.edu.tr; 6 Department of Medicinal Chemistry, Faculty of Pharmacy, Mansoura University, Mansoura 35516, Egypt/; 7 Department of Chemistry, Faculty of Science, Mansoura University, Mansoura 35516, Egypt; biobotany@gmail.com

**Keywords:** chalcone, indole, crystal structure, cytotoxic activity

## Abstract

The crystal structures of five new chalcones derived from *N*-ethyl-3-acetylindole with different substituents were investigated: (*E*)-3-(4-bromophenyl)-1-(1-ethyl-1*H*-indol-3-yl)prop-2-en-1-one (**3a**); (*E*)-3-(3-bromophenyl)-1-(1-ethyl-1*H*-indol-3-yl)prop-2-en-1-one (**3b**); (*E*)-1-(1-ethyl-1*H*-indol-3-yl)-3-(4-methoxyphenyl)prop-2-en-1-one (**3c**); (*E*)-1-(1-ethyl-1*H*-indol-3-yl)-3-mesitylprop-2-en-1-one (**3d**); and (*E*)-1-(1-ethyl-1*H*-indol-3-yl)-3-(furan-2-yl)prop-2-en-1-one (**3e**). The molecular packing of the studied compounds is controlled mainly by C–H⋅⋅⋅O hydrogen bonds, C–H⋅⋅⋅π interactions, and π···π stacking interactions, which were quantitatively analyzed using Hirshfeld topology analysis. Using density functional theory (DFT) calculations, the order of polarity (**3b ˂ 3d ˂ 3e ˂ 3a ˂ 3c**) was determined. Several chemical reactivity indices such as the ionization potential (I), electron affinity (A), chemical potential (μ), hardness (η), electrophilicity (ω) and nucleophilicity (N) indices were calculated, and these properties are discussed and compared. In addition, the antiproliferative activity of the five new chalcones was studied.

## 1. Introduction

The class of compounds based on an indole scaffold is of immense chemical and biological significance [1,2]. Among the nitrogen containing heterocycles, indole is the parent core in a large number of naturally occurring bioactive compounds. Indole and its derivatives have received significant attention because of their wide range of biological activities including antimicrobial, anticancer, anti-HIV, antileishmanial, and anti-inflammatory activities [3]. Many natural, as well as synthetic, indole analogs are potent anticancer agents. Recently, Reyes and coworkers isolated a family of indole alkaloids, aplicyanins, from the Antarctic tunicate *Aplidiumcyaneum* [4]. The aplicyanins with a bromoindole nucleus and 6-tetrahydropyrimidine at the C-3 position were found to be cytotoxic against human tumor cell lines, including MDA-MB-231 (breast adenocarcinoma), A549 (lung carcinoma), and HT-29 (colorectal carcinoma), via the inhibition of tubulin polymerization [5,6,7,8,9,10]. Hu et al. reported indolylpyrimidine as a potent antiproliferative agent with average 50% inhibitory concentration (IC_50_) values ranging from 5.01 to 14.36 µM against the tested cancer cell lines [11].

After heart disease, cancer is the prominent cause of deaths worldwide, and the identification of potent and selective anticancer drugs with reduced side effects requires increasingly sophisticated techniques in medicinal chemistry. Here, we report the synthesis, X-ray single crystal, DFT, and Hirshfeld surface analysis. The selective anticancer activity of some indole-chalcone-based compounds were evaluated using seven cancer cell lines and proved that ER-negative breast cancer cells (MDA-MB-231) were more sensitive to some of the tested compounds than the ER-positive breast cancer cell line (MCF-7).

## 2. Results

### Synthesis

**Scheme 1:**
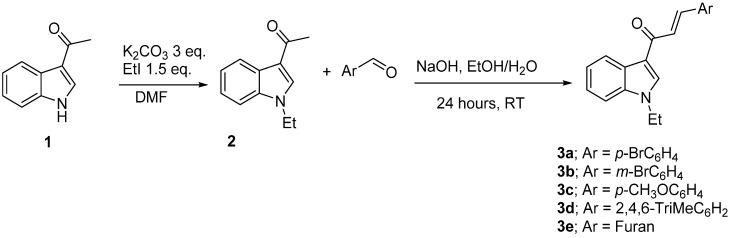
Synthesis of chalcones (*E*)-3-(4-bromophenyl)-1-(1-ethyl-1*H*-indol-3-yl)prop-2-en-1-one (**3a**); (*E*)-3-(3-bromophenyl)-1-(1-ethyl-1*H*-indol-3-yl)prop-2-en-1-one (**3b**); (*E*)-1-(1-ethyl-1*H*-indol-3-yl)-3-(4-methoxyphenyl)prop-2-en-1-one(**3c**); (*E*)-1-(1-ethyl-1*H*-indol-3-yl)-3-mesitylprop-2-en-1-one(**3d**); and (*E*)-1-(1-ethyl-1*H*-indol-3-yl)-3-(furan-2-yl)prop-2-en-1-one (**3e**).

The requisite compounds chalcones were prepared by the reaction of *N*-ethyl-3-acetylindole and aryl aldehyde derivatives with stirring in EtOH/H_2_O (1:1) with NaOH at room temperature for 24 h. The product was produced in high yield (up to 90%), as shown in Scheme 1.

## 3. Discussion

### 3.1. X-ray Structure

**Figure 1:**
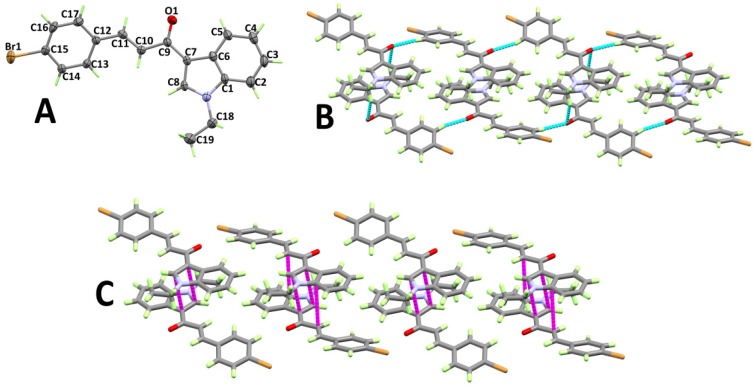
Atom numbering and thermal ellipsoids drawn at 30% probability level (**A**), hydrogen bonds (**B**), and π–π stacking interactions (**C**) in 3a.

The structure and atom numbering of (*E*)-3-(4-bromophenyl)-1-(1-ethyl-1*H*-indol-3-yl)prop-2-en-1-one (**3a**) are shown in Figure 1A. Having four molecular units per unit cell and one molecule per asymmetric unit, **3a** crystallized in the monoclinic crystal system and the *P*2_1_/*c* space group. The most important bond distances and angles are given in Table S2 (Supplementary data). The indole ring is perfectly planar, and the *p*-bromophenyl moiety deviates by 13.21° from the mean plane of the indole ring. The molecules are packed in the crystal structure via weak C–H⋅⋅⋅O hydrogen bonds (Figure 1B) and the π–π stacking interactions, as shown in Figure 1C. Details regarding the hydrogen bond parameters are given in Table 1. The shortest C⋅⋅⋅C contact distances among the stacked indole rings are 3.408 and 3.484 Å for the C7⋅⋅⋅C8 and C1⋅⋅⋅C10 contacts, respectively.

**Figure 2:**
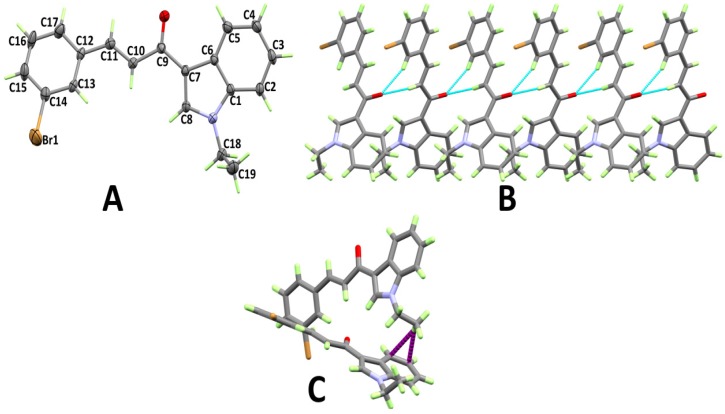
Atom numbering and thermal ellipsoids drawn at 30% probability level (**A**), hydrogen bonds, (**B**) and C–H···π interactions (**C**) in **3b**.

The structure and atom numbering of (*E*)-3-(3-bromophenyl)-1-(1-ethyl-1*H*-indol-3-yl)prop-2-en-1-one (**3b**) are shown in Figure 2A. Having four molecular units per unit cell and one molecule per asymmetric unit, **3b** crystallized in the monoclinic crystal system and the *P*2_1_/*c* space group. The most important bond distances and angles are given in Table S2 (Supplementary data). The *m*-bromophenyl moiety deviates from the mean plane of the indole ring more than that in **3a**, probably because of the presence of the bulky Br atom at the *meta* position. The twist angle between the mean plane of the indole ring and the *m*-bromophenyl moiety is 25.96°. The molecules are packed in the crystal structure via weak C–H⋅⋅⋅O hydrogen bonds and C–H⋅⋅⋅π interactions, as shown in Figure 2B,C, respectively. C–H⋅⋅⋅O hydrogen bonds occur between the carbonyl oxygen atom as the hydrogen bond acceptor and the neighboring C–H groups as the hydrogen bond donors (Table 1). The C–H···π interactions occurred between the C–H hydrogen atoms from the methyl group and the carbon atoms from the phenyl group of the indole moiety. The shortest C–H···π contact distances are 2.992 and 2.993 Å for the C4⋅⋅⋅H19B and C5⋅⋅⋅H19B contacts, respectively.

**Figure 3:**
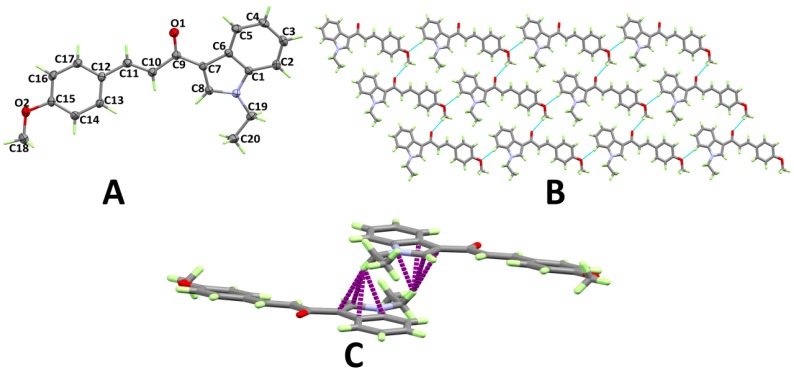
Atom numbering and thermal ellipsoids drawn at 30% probability (**A**), hydrogen bonds (**B**), and C–H···π interactions (**C**) in **3c**.

The structure and atom numbering of (*E*)-1-(1-ethyl-1*H*-indol-3-yl)-3-(4-methoxyphenyl)prop-2-en-1-one (**3c**) are shown in Figure 3A. In this case, compound **3c** crystallized in the triclinic crystal system and the *P*-1 space group, having two molecular units per unit cell and one molecule per asymmetric unit. The most important bond distances and angles are given in Table S2 (Supplementary data). In this case, the *p*-methoxyphenyl group showed smaller twist (12.20°) from the mean plane of the indole ring compared to those in compounds **3a** and **3b**. The methoxy group O-atom as a hydrogen acceptor site connects the molecules via C–H⋅⋅⋅O hydrogen bonds with the neighboring C–H bonds (Figure 3B). In addition, the indole rings are connected by C–H⋅⋅⋅π interactions between one hydrogen atom from the CH_2_ group with the four C-atoms in the indole five-membered ring, as shown in Figure 3C. The C–H···π contact distances range from 2.909 Å (C7⋅⋅⋅H19B) to 2.980 Å (C1⋅⋅⋅H19B).

**Figure 4:**
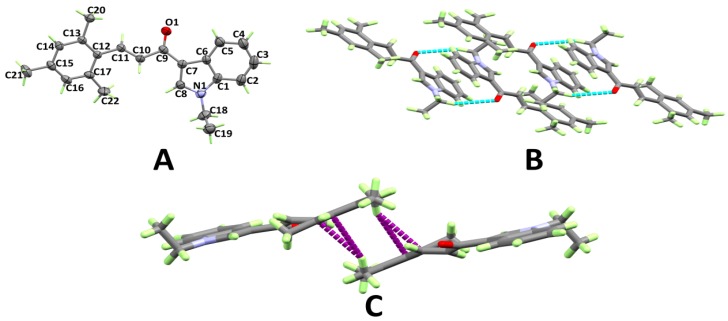
Atom numbering and thermal ellipsoids drawn at 30% probability level (**A**), hydrogen bonds (**B**), and C–H···π (**C**) stacking interactions in **3d**.

The structure and atom numbering of (*E*)-1-(1-ethyl-1*H*-indol-3-yl)-3-mesitylprop-2-en-1-one (**3d**) are shown in Figure 4A. Like **3c**, this compound crystallized in the triclinic crystal system and the *P*-1 space group, having two molecular units per unit cell and one molecule per asymmetric unit. The most important bond distances and angles are given in Table S2 (Supplementary data). In this case, the mesitylene ring showed a similar twist from the mean plane of the indole ring to that of **3c** (11.98°). In this case, the carbonyl group O-atom is the hydrogen bond acceptor site and one C–H bond from the CH_2_ group connect the molecules via C–H···O hydrogen bonds (Table 1), thus forming disconnected dimers of **3d**, as shown in Figure 4B. In addition, the molecules are connected by C–H···π interactions between the stacked aryl moieties. The hydrogen atoms from the methyl substituents at the aryl moiety form C–H···π contacts with the aromatic π-system from another aryl moiety (Figure 4C). The C–H···π contact distances range from 2.914 Å (C16···H20B) to 2.934 Å (C17···H20B).

**Figure 5:**
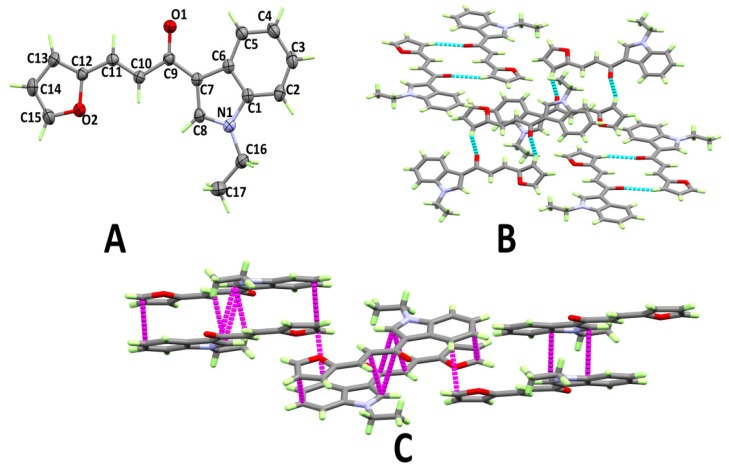
Atom numbering and thermal ellipsoids drawn at 30% probability level (**A**), hydrogen bonds (**B**), and π···π (**C**) stacking interactions in **3e**.

The structure and atom numbering of (*E*)-1-(1-ethyl-1*H*-indol-3-yl)-3-(furan-2-yl)prop-2-en-1-one (**3e**) are shown in Figure 5A. Having four molecular units per unit cell and one molecule per asymmetric unit, **3e** crystallized in the monoclinic crystal system and the *P*2_1_/*c* space group. The most important bond distances and angles are given in Table S2 (Supplementary data). In this case, the furanyl ring showed the least twist from the mean plane of the indole ring (3.47°) of the studied compounds. The carbonyl group O-atom and one C–H bond from the furanyl ring connect the molecules via C–H···O hydrogen bonds, forming disconnected dimers of **3e** (Figure 5B). Details regarding the hydrogen bond parameters are given in Table 1. In addition, the molecules are connected by π···π stacking interactions between the stacked molecular units. The C···C contact distances ranges from 3.437 Å (C8···C10) to 3.496 Å (C8···C9) (Figure 5C).

### 3.2. Analysis of Molecular Packing

**Figure 6:**
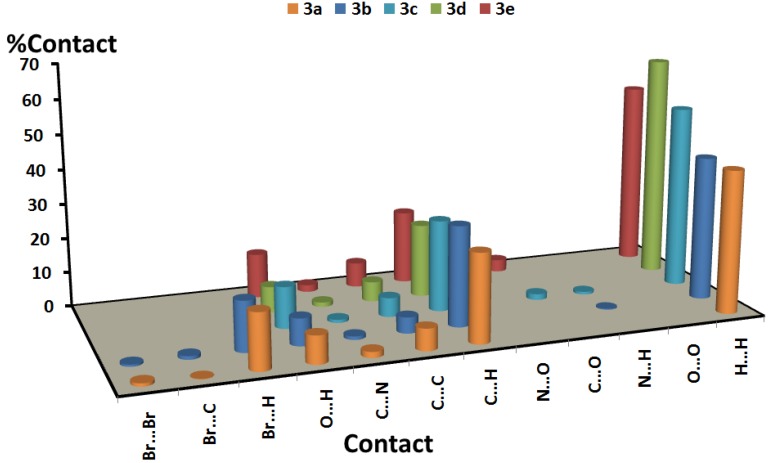
Summary of the intermolecular interactions and their percentages in the crystal structures of the studied compounds.

**Figure 7:**
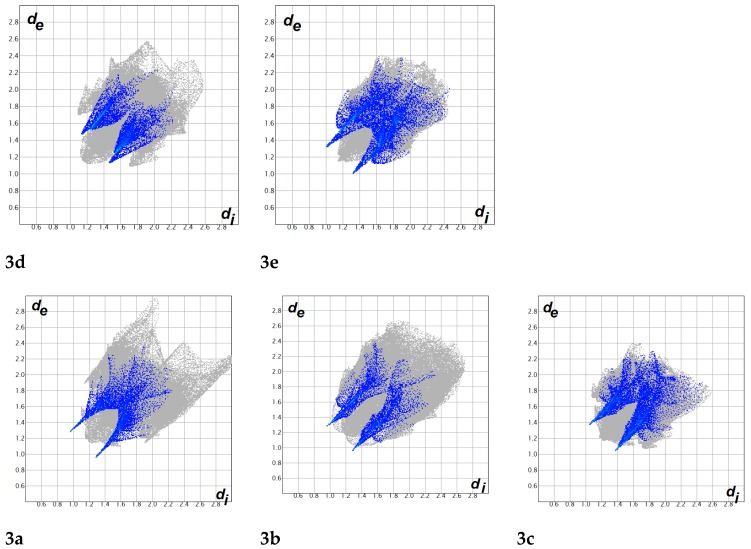
The decomposed fingerprint (FP) plots of the O···H contacts in the studied compounds: Sharper spikes indicate shorter O···H contacts. The O···H contacts are the shortest in compound **3a** and the longest in **3d**.

**Figure 8:**
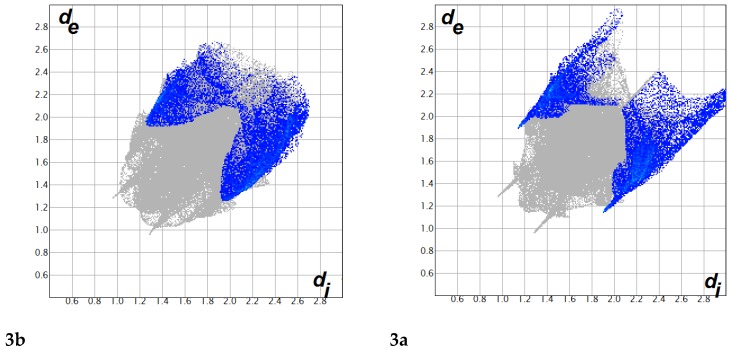
The decomposed FP plots of the Br···H contacts in compounds **3b** (left) and **3a** (right): The sharper spikes in the FP plot of compound **3a** indicate shorter contact distances compared to **3b**.

Hirshfeld surfaces mapped over *d*_norm_, the shape index (SI), and the curvedness for the studied compounds are shown in Figure S1 (Supplementary data). A summary of the most important contacts and their percentages are shown in Figure 6 and Table 2, respectively. All the studied systems contained many H···H, O···H, and C···H contacts. The H···H contacts range from 41.4% (compound **3a**) to 64.4% (compound **3d**), and the percentage of O···H hydrogen bonds is highest in compound **3e** (12.8%) and is lowest in compound **3b** (7.8%). Most of these hydrogen bonds are considered short contacts and are important in the molecular packing of all the studied compounds, as indicated from the sharp spikes of these interactions in the corresponding fingerprint plots (see Figure 7). The strongest hydrogen bond was observed in compound **3a** (2.243 Å), whereas the weakest was observed in compound **3d** (2.601 Å). The two bromo-substituted compounds form many short Br···H interactions, ranging from 14.4% (compound **3b**) to 16.0% (compound **3a**). It is clear from Figure 8 that the Br···H interactions are more significant in the latter (3.045 Å, Br1···H2A) compared to the former (3.203 Å, Br1···H19A). Most of the studied compounds show significant π···π stacking interactions as well as C–H···π stacking interactions. The percentages of the C···C/C···N contacts range from 5.6% (compound **3b**) to 8.0% (compound **3a**). Those π···π stacking interactions appeared as blue/red triangles in the shape index maps of the studied molecules. On the other hand, the C···H contact percentages ranges from 21.1% (compounds **3d** and **3e**) to 28.0% (compound **3b**). These appear in the shape index maps as a large red hole, which is related to the aromatic π-systems involved in the C–H···π interactions.

### 3.3. Reactivity Studies

The reactivity indices of the studied compounds (ionization potential (I), electron affinity (A), chemical potential (μ), hardness (η), softness (S), electronegtivity (x), electrophilicity (ω) and nucleophilicity (N) indices were calculated using Equations (1)–(6) [12,13,14,15,16,17,18,19,20], and the results are listed in Table 3. These descriptors were used to understand the pharmacological and biological reactivity of the compounds.

I = −E_HOMO_, (1)

A = −E_LUMO_, (2)

η = (I − A),(3)

μ = −(I + A)/2,(4)

ω = μ^2^/2η, (5)

N = E_HOMO_(Nucleophile) − E_HOMO_(TCE) (6)

The results show that compound **3d** is the hardest molecule while **3b** is the softest. Based on the chemical potentials, the most electron donating system is compound **3d**. In agreement with this conclusion, the electrophilicity index of the studied compounds decreases in the order **3b** > **3a** > **3e** > **3d** > **3c**. In addition, **3b** has the highest electrophilicity index [19], which is consistent with the presence of a strong electron-withdrawing substituent on the phenyl ring.

The nucleophilicity N index [20] is referred to tetracyanoethylene (TCE), which is the most electrophilic neutral species. Hence, the expected least nucleophilic neutral species which allowed convenient handling of a nucleophilicity scale of positive values. It is clear that, the order of the compounds according to the global nucleophilicity index is **3c** > **3e** > **3d** > **3a** > **3b**. Among the studied compounds, 3b has the highest nucleophilic characters among the others.

### 3.4. Biological Activity

The anticancer activities of the synthesized chalcone derivatives were tested against seven cancer cell lines: ER-positive MCF-7 and triple negative MDA-MB-231 breast cancer cells, liver cancer (HepG2 and HuH-7), prostate cancer (PC-3), colon cancer (HCT-116), and oral cancer (SAS) (Table 4). The triple negative breast cancer cells (MDA-MB-231) were more sensitive to the tested compounds (**3a**–**e**) than the ER-positive breast cancer cell line (MCF-7), where their IC_50_ values ranged from 13 to 19 µM, which is comparable to that of cisplatin (15 µM) (Table 4). Compounds **3b**, **3d**, and **3e** showed moderate anticancer activity only against HepG2 and not the other hepatocellular carcinoma cell line (HuH-7), unlike cisplatin, which was active against both liver cancer cell lines. Compounds **3c** and **3d** did not show cytotoxic activity against the oral, prostate or colon cancer cell lines.

## 4. Materials and Methods

### 4.1. General Procedure for the Synthesis of Chalcones 3a–e

The chalcones were prepared following a reported procedure [21,22,23].

*(E)-3-(4-Bromophenyl)-1-(1-ethyl-1H-indol-3-yl)prop-2-en-1-one (3a)* [22,23]. Yield 1.75 g (4.95 mmol, 93%); m.p. 139–140 °C; ^1^H-NMR (400 MHz, DMSO-*d*_6_) *δ*: 1.49 (t, 3H, *J* = 7.2 Hz, CH_3_), 4.17 (q, 3H, *J* = 7.2 Hz, CH_2_), 7.23–7.29 (m, 4H, Ar–H and CH=CH), 7.41 (q, 4H, *J* = 6.8 Hz, Ar–H), 7.64 (d, 1H, *J* = 15.2 Hz, CH=CH), 7.82 (s, 1H, Ar–H), 8.42–8.45 (m, 1H, Ar–H); ^13^C-NMR (100 MHz, DMSO-*d*_6_) *δ*: 15.1, 41.8, 109.8, 117.6, 122.7, 123.0, 123.6, 123.8, 124.4, 126.9, 129.5, 131.9, 133.7, 133.3, 136.7, 139.5, and 183.8; IR (KBr, cm^−1^) ${\bar{v}}_{\max}$ = 3449, 3041, 2977, 1645, 1610, 1588, 1525, 1481, 1469, 1454, 1399, 1310, 1241, 1267, 1268, 1016, 978, 835, 771, 744, 718, 675, and 478; Anal. Calcd. for C_19_H_16_BrNO: C, 64.42; H, 4.55; N, 3.95; Found: C, 64.31; H, 4.67; N, 4.15; Liquid chromatography (LC)/mass spectrometry (MS) (electrospray ionization (ESI), *m*/*z*): [M^+^], found 353.00, C_19_H_16_BrNO for 353.04.

*(E)-3-(3-Bromophenyl)-1-(1-ethyl-1H-indol-3-yl)prop-2-en-1-one (3b)* [23]. Yield 1.60 g (4.53 mmol, 85%); m.p. 126–127 °C; ^1^H-NMR (400 MHz, DMSO-*d*_6_) *δ*: 1.54 (t, 3H, *J* = 5.6 Hz, CH_3_), 4.22 (q, 2H, *J* = 5.6 Hz, CH_2_), 7.25–7.36 (m, 5H, Ar–H and CH=CH), 7.49 (t, 2H, *J* = 8.8 Hz, Ar–H), 7.68–7.78 (m, 2H, *J* = 15.2 Hz, Ar–H and CH=CH), 7.92 (s, 1H, Ar–H), 8.50–8.53 (m, 1H, Ar–H); ^13^C-NMR (100 MHz, DMSO-*d*_6_) *δ*: 15.3, 42.0, 109.9, 117.7, 122.9, 123.01, 123.2, 123.8, 125.1, 127.05, 127.3, 130.3,130.4, 132.6, 134.03, 136.8, 137.6, 139.3, and 183.7; IR (KBr, cm^−1^) ${\bar{v}}_{\max}$ = 3445, 3042, 2971, 1643, 1617, 1585, 1521, 1482, 1467, 1455, 1399, 1312, 1242, 1261, 1264, 1012, 976, 837, 777, 744, 715, 673, and 474; Anal. Calcd. for C_19_H_16_BrNO: C, 64.42; H, 4.55 N, 3.95; found: C, 64.65; H, 4.38; N, 4.17; LC/MS (ESI, *m*/*z*): [M^+^ found 353.00, C_19_H_16_BrNO for 353.04.

*(E)-1-(1-Ethyl-1H-indol-3-yl)-3-(4-methoxyphenyl)prop-2-en-1-one (3c)* [23]. Yield 1.60 g (5.2 mmol, 98%); m.p. 123–124 °C; ^1^H-NMR (400 MHz, DMSO-*d*_6_) *δ*: 1.52 (t, 3H, *J* = 6.4 Hz, CH_3_), 3.82 (s, 3H, OCH_3_) 4.18 (q, 2H, *J* = 7.2 Hz, CH_2_), 6.88 (d, 1H, *J* = 8.8 Hz, Ar–H), 7.2–7.33 (m, 4H, *J* = 8.4 Hz, Ar–H and CH=CH), 7.55 (d, 2H, *J* = 8.8 Hz, Ar–H), 7.76 (d, 1H, *J* = 15.2 Hz, Ar–H), 7.87 (s, 1H, Ar–H), 8.50–8.52 (m, 1H, Ar–H); ^13^C-NMR (100 MHz, DMSO-*d*_6_) *δ*: 15.1, 41.7, 55.3, 109.6, 114.6, 117.7, 121.6, 122.4, 123.0, 123.3, 127.0, 128.0, 129.7, 133.4, 136.6, 140.6, 161.0, and 184.4; IR (KBr, cm^−1^) ${\bar{v}}_{\max}$ = 3431, 3104, 2988, 2947, 2832, 1749, 1679, 1645, 1589, 1527, 1514, 1426, 1377, 1332, 1243, 1207, 1125, 1108, 1006, 844, and 755; Anal. Calcd. for C_20_H_19_NO_2_: C, 78.66; H, 6.27; N, 4.59; found: C, 78.85; H, 6.12; N, 4.85; LC/MS (ESI, *m*/*z*): [M^+^], found 305.10, C_20_H_19_NO_2_ for 305.38.

*(E)-1-(1-Ethyl-1H-indol-3-yl)-3-mesitylprop-2-en-1-one (3d)* [23]. Yield 1.10 g (3.4 mmol, 65%); m.p. 83–84 °C; ^1^H-NMR (400 MHz, DMSO-*d*_6_) *δ*: 1.52 (t, 3H, *J* = 7.6 Hz, CH_3_), 2.29 (s, 3H, CH_3_) 2.36 (s, 6H, CH_3_), 4.22 (q, 2H, *J* = 7.6 Hz, CH_2_), 6.91 (s, 2H, Ar–H), 6.99 (d, 1H, *J* = 16.4 Hz, CH=CH), 7.30–7.38 (m, 3H, Ar–H), 7.80 (s, 1H, Ar–H), 7.93 (d, 1H, *J* = 16.4 Hz, CH=CH), 8.50–8.53 (m, 1H, Ar–H); ^13^C-NMR (100 MHz, DMSO-*d*_6_) *δ*: 15.3, 21.1, 21.3, 41.9, 109.8, 117.8, 122.7, 123.2, 123.6, 127.1, 129.1, 129.3, 132.3, 133.8, 136.8, 136.9, 137.9, 139.6, and 184.5; IR (KBr, cm^−1^) ${\bar{v}}_{\max}$ = 3041, 2974, 1641, 1584, 1525, 1483, 1447, 1385, 1302, 1294, 1202, 1206, 1188, 1089, 1062, 992, 978, 862, 816, 772, 725, 601, 495, 449, and 422; Anal. Calcd. for C_22_H_23_NO: C, 83.24; H, 7.30; N, 4.41; found: C, 83.52; H, 7.19; N, 4.61; LC/MS (ESI, *m*/*z*): [M^+^], found 317.20; C_22_H_23_NO for 317.18.

*(E)-1-(1-Ethyl-1H-indol-3-yl)-3-(furan-2-yl)prop-2-en-1-one (3e)* [23]. Yield 1.35 g (5.09 mmol, 95%); m.p. 74–75 °C; ^1^H-NMR (400 MHz, DMSO-*d*_6_) *δ*: 1.54 (t, 3H, *J* = 7.2 Hz, CH_3_), 4.23 (q, 2H, *J* = 7.2 Hz, CH_2_), 7.32–7.37 (m, 3H, Ar–H), 7.42 (d, 1H, *J* = 15.6 Hz, CH=CH), 7.61 (d, 2H, *J* = 8.0 Hz, Ar–H), 7.69 (d, 2H, *J* = 8.0 Hz, Ar–H), 7.75 (d, 1H, *J* = 15.2 Hz, CH=CH), 7.92 (s, 1H, Ar–H), 8.49–8.51 (m, 1H, Ar–H); ^13^C-NMR (100 MHz, DMSO-*d*_6_) *δ*: 15.1, 41.7, 109.7, 112.3, 114.6, 117.7, 121.3, 123.0, 123.4, 126.9, 127.4, 133.8, 136.7, 144.0, 152.0, and 183.7; IR (KBr, cm^−1^) ${\bar{v}}_{\max}$ = 3478, 3102, 3075, 2972, 2929, 1631, 1562, 1524, 1488, 1442, 1389, 1365, 1204, 1102, 1086, 964, 857, 832, 744, 578, and 653; Anal. Calcd. for C_17_H_15_NO_2_: C, 76.96; H, 5.70; N, 5.28; found: C, 76.55; H, 5.95; N, 5.10; LC/MS (ESI, *m*/*z*): [M^+^], found 265.10, C_17_H_15_NO_2_ for 265.11.

### 4.2. X-ray Structure Measurements

X-ray crystal data were collected on a Bruker APEX-II D8 Venture area diffractometer. Crystal data, data collection, and structure refinement details are summarized in Table S1 (Supplementary data). All H atoms were found in the difference Fourier maps.

### 4.3. DFT Calculations

The structures of the studied systems were optimized at the B3LYP/6-311G (d,p) level of theory using Gaussian 09 [24]. In all cases, the input structures were taken from the CIF data obtained from the corresponding X-ray single crystal structures. All optimized structures showed no imaginary vibrations in the frequency calculations. All images were prepared in GaussView [25].

### 4.4. Hirshfeld Surface Analysis

The topology analyses were performed using Crystal Explorer 3.1 [26] to determine the percentages of different intermolecular interactions in the crystal structure of the studied complexes.

### 4.5. Anticancer Activity Assays

The anticancer activities for the five compounds were evaluated against five different cancer types (breast, oral, prostate, colon, and liver). The initial screening was performed at 50 µM, and the inhibitory activity was detected using the crystal violet assay [27]. Subsequently, the compound that showed inhibitory activity was further used in MTT assay for determination of the IC_50_ [28]. The standard deviation (±) was calculated from three independent experiments.

## 5. Conclusions

Five chalcone-based indole scaffolds were successfully synthesized from *N*-alkyl-3-acetylindole and five aldehydes. Structures of the obtained chalcones were clarified, and the study of the chemical behavior including (DFT) calculations, ionization potential (I), electron affinity (A), chemical potential (μ), hardness (η), electrophilicity (ω) and nucleophilicity (N) indices and Hirshfeld surface analysis were investigated. The anticancer activity of the synthesized chalcones was carried out and exhibited more sensitivity to the triple negative breast cancer cells (MDA-MB-231) than the ER-positive breast cancer cell line (MCF-7). Further investigation will be carried out in the near future.

## Figures and Tables

**Table 1:** Hydrogen bond parameters in the studied compounds.

D–H···A	D–H (Å)	H···A (Å)	D···A (Å)	D–H···A (°)	Symm. Code^i^
**3a**					
C14–H14A···O1^i^	0.930	2.390	3.307 (7)	167.7	*x*, 3/2 − *y*, −1/2 + *z*
C18–H18B···O1^i^	0.971	2.733	3.420 (1)	128.5	1 − *x*, 2 − *y*, 1 − *z*
**3b**					
C10–H10A···O1^i^	0.931	2.526	3.447 (5)	170.8	*x*, 3/2 − *y*, −1/2 + *z*
C13–H13A···O1^i^	0.931	2.391	3.269 (5)	157.1	*x*, 3/2 − *y*, −1/2 + *z*
**3c**					
C2–H2A···O2^i^	0.930	2.562	3.474 (2)	166.8	−1 + *x*, −1 + *y*, 1 + *z*
C18–H18B···O1^i^	0.960	2.617	3.441 (2)	144.1	*x*, −1 + *y*, *z*
**3d**					
C18–H18B···O1^i^	0.971	2.697	3.564 (5)	149.0	−*x*, 1 − *y*, 1 − *z*
**3e**					
C13–H13A···O1^i^	0.930	2.460	3.291 (3)	148.8	1 − *x*, 2 − *y*, 1 − *z*

**Table 2:** List of all intermolecular contacts and their percentages using Hirshfeld Topology analysis.

	3a	3b	3c	3d	3e
**Br…Br**	0.9	0.6			
**Br…C**	0.1	1.0			
**Br…H**	16.0	14.4			
**O…H**	8.0	7.8	12.0	7.5	12.8
**C…N**	1.7	1.0	0.9	1.3	2.0
**C…C**	6.3	4.6	5.5	5.7	7.1
**C…H**	25.5	28.8	26.3	21.1	21.1
**N…O**					
**C…O**			1.7		3.5
**N…H**		0.3	0.8		
**O…O**					
**H…H**	41.4	41.5	52.8	64.4	53.5

**Table 3:** The calculated dipole moment (μ), *E*_HOMO_, and *E*_LUMO_ and the calculated reactivity indices (eV) of the studied compounds calculated using B3LYP/6-311G(d,p) method.

	3a	3b	3c	3d	3e
μ	5.650	3.866	7.282	5.352	5.573
*E*_HOMO_	−5.909	−5.949	−5.676	−5.812	−5.730
*E*_LUMO_	−2.132	−2.175	−1.758	−1.771	−1.887
Δ*E*	3.777	3.773	3.918	4.041	3.843
I	5.91	5.95	5.68	5.81	5.73
A	2.13	2.18	1.76	1.77	1.89
η	3.78	3.77	3.92	4.04	3.84
μ	-4.02	-4.06	-3.72	-3.79	-3.81
x	4.02	4.06	3.72	3.79	3.81
S	0.26	0.27	0.26	0.25	0.26
ω	2.14	2.19	1.76	1.78	1.89
N	3.46	3.42	3.69	3.56	3.64

**Table 4:** IC_50_ values of the tested chalcone derivatives against seven cancer cell lines: The symbol ± represents the standard deviation from three independent experiments. The viability of the cells was detected using the MTT assay. NA means that the compound did not show cytotoxic activity at 50 µM.

Type	Breast		Oral	Prostate	Colon	Liver	
Cell line	MCF-7	MDA-MB-231	SAS	PC-3	HCT-116	HuH-7	HepG2
**3a**	30 ± 2	15 ± 1	30 ± 1.5	30 ± 2.2	20 ± 1.3	28.4 ± 1.67	20 ± 1.5
**3b**	>50	13 ± 0.5	29 ± 3	>50	25 ± 2.1	NA	15 ± 0.57
**3c**	>50	19 ± 1.6	NA	NA	NA	NA	25 ± 0.97
**3d**	50 ± 5.5	18.5 ± 1	NA	NA	NA	NA	25 ± 1.7
**3e**	35 ± 3	18 ± 2.4	32 ± 2	42 ± 3	30 ± 2.5	29.2 ± 3.4	29 ± 1.4
***cis*platin**	9 ± 0.3	15 ± 1.6	4.5 ± 0.4	12 ± 1	8 ± 1.3	14.7 ± 2	10 ± 1.2

## Supplementary Materials

The following are available online. Table S1: Crystal data and refinement details; Table S2: Geometric parameters (bond distances (Å) and angles (°)) of the studied compounds; Table S3: The calculated geometric parameters (bond distances and angles) of the studied compounds; Figure S1: The optimized molecular structures of the studied molecules.
