# Supplementary material for: Anticancer Indole-Based Chalcones: A Structural and Theoretical Analysis
Source: Molecules. 2019 Oct 16;24(20):3728. doi: 10.3390/molecules24203728 (PMC6832658; doi:10.3390/molecules24203728)
Supplement: Supplementary file 1 [file molecules-24-03728-s001.pdf]

Table S1. Crystal data and refinement details.

	**3a**	**3b**	**3c**	**3d**	**3e**
Empirical formula	$C_{19}H_{16}BrNO$	$C_{19}H_{16}BrNO$	$C_{20}H_{19}NO_2$	$C_{22}H_{23}NO$	$C_{17}H_{15}NO_2$
Formula weight	354.24	354.24	305.36	317.41	265.3
Temperature/K					
Crystal system	monoclinic	monoclinic	triclinic	triclinic	monoclinic
Space group	$P2_1/c$	$P2_1/c$	P-1	P-1	$P2_1/c$
a/Å	12.8036(12)	14.128(7)	7.7513(7)	7.708(3)	7.9921(3)
b/Å	6.8390(6)	13.354(6)	9.3534(7)	8.992(4)	9.2286(5)
c/Å	18.561(2)	8.823(4)	11.0314(10)	13.965(6)	18.6079(9)
α/°	90	90	83.927(3)	107.405(12)	90
β/°	94.525(3)	103.575(12)	82.172(3)	96.774(13)	96.8750(10)
γ/°	90	90	87.817(3)	98.407(12)	90
Volume/Å^3	1620.2(3)	1618.1(13)	787.66(12)	900.1(6)	1362.57(11)
Z	4	4	2	2	4
ρ_{calc}g/cm^3	1.452	1.454	1.288	1.171	1.293
μ/mm^{-1}	2.538	2.541	0.083	0.071	0.085
F(000)	720	720	324	340	560
Crystal size/mm^3	0.317 × 0.269 × 0.085	0.418 × 0.159 × 0.09	0.417 × 0.264 × 0.225	0.520 × 0.419 × 0.115	0.444 × 0.315 × 0.226
Radiation					
2Θ range	6.352 to 55	5.646 to 54.998 °	4.38 to 53.998	5.768 to 54.998	4.934 to 50
Index ranges	−16 ≤ h ≤ 16, −8 ≤ k ≤ 8, −23 ≤ l ≤ 24	−18 ≤ h ≤ 17, −17 ≤ k ≤ 17, −11 ≤ l ≤ 11	−9 ≤ h ≤ 9, −10 ≤ k ≤ 11, −14 ≤ l ≤ 14	−10 ≤ h ≤ 10, −11 ≤ k ≤ 11, −17 ≤ l ≤ 18	−9 ≤ h ≤ 9, −10 ≤ k ≤ 10, −22 ≤ l ≤ 22

Reflections collected	20574	23959	19525	14528	49011
Independent reflections	3720 (R_{int} = 0.1464, R_{sigma} = 0.1154)	3710 (R_{int} = 0.1452, R_{sigma} = 0.0990)	3428 (R_{int} = 0.0679, R_{sigma} = 0.0486)	4111 (R_{int} = 0.1376, R_{sigma} = 0.1533)	2398 (R_{int} = 0.1455, R_{sigma} = 0.0394)
Data/restraints/parameters	3720/0/200	3710/0/200	3428/0/208	4111/0/222	2398/0/183
Goodness-of-fit on F^2	0.978	0.962	1.05	0.999	1.078
Final R indexes ($I \geq 2\sigma$ (I))	R_1 = 0.0834, wR_2 = 0.2015	R_1 = 0.0539, wR_2 = 0.1003	R_1 = 0.0513, wR_2 = 0.1244	R_1 = 0.0825, wR_2 = 0.2050	R_1 = 0.0518, wR_2 = 0.1242
Final R indexes (all data)	R_1 = 0.1782, wR_2 = 0.2668	R_1 = 0.1489, wR_2 = 0.1320	R_1 = 0.0888, wR_2 = 0.1449	R_1 = 0.1949, wR_2 = 0.2610	R_1 = 0.0714, wR_2 = 0.1401
Largest diff. peak/hole/e $Å^{-3}$	2.39/−0.67	0.48/−0.47	0.22/−0.31	0.24/−0.18	0.24/−0.17

Table S2. Geometric parameters (bond distances (Å) and angles (°)) of the studied compounds.

**3a**		**3b**		**3c**		**3d**		**3d**	
Br1–C15	1.904(7)	Br1–C14	1.883(4)	O1–C9	1.230(2)	O1–C9	1.227(5)	O1–C9	1.236(2)
N1–C18	1.473(8)	O1–C9	1.228(4)	N1–C8	1.3503(19)	C1–C2	1.388(5)	O2–C15	1.358(3)
C3–C4	1.376(12)	N1–C8	1.344(4)	C2–C3	1.371(2)	C4–C5	1.380(6)	O2–C12	1.367(2)
C7–C8	1.383(8)	N1–C1	1.391(4)	C6–C7	1.439(2)	C7–C9	1.442(5)	N1–C8	1.354(2)
C11–C12	1.459(8)	N1–C18	1.460(5)	C10–C11	1.329(2)	C12–C13	1.395(4)	N1–C1	1.386(3)
C14–C15	1.365(9)	C1–C6	1.391(5)	C13–C14	1.381(2)	C14–C15	1.390(5)	N1–C16	1.460(3)
O1–C9	1.231(7)	C1–C2	1.395(5)	C19–C20	1.497(2)	C17–C22	1.510(5)	C1–C2	1.393(3)
C1–C2	1.401(10)	C2–C3	1.377(5)	O2–C15	1.370(2)	N1–C1	1.384(5)	C1–C6	1.400(3)
C4–C5	1.382(10)	C3–C4	1.381(6)	N1–C19	1.470(2)	C1–C6	1.390(4)	C2–C3	1.367(3)
C7–C9	1.451(8)	C4–C5	1.381(5)	C3–C4	1.390(3)	C5–C6	1.410(5)	C3–C4	1.379(4)
C12–C13	1.400(8)	C5–C6	1.393(5)	C7–C8	1.375(2)	C9–C10	1.486(5)	C4–C5	1.376(3)
C15–C16	1.363(10)	C6–C7	1.445(5)	C11–C12	1.458(2)	C12–C17	1.415(4)	C5–C6	1.399(3)
N1–C1	1.375(8)	C7–C8	1.373(5)	C14–C15	1.386(2)	C15–C16	1.361(6)	C6–C7	1.447(3)
C1–C6	1.384(9)	C7–C9	1.446(5)	O2–C18	1.417(2)	C18–C19	1.469(6)	C7–C8	1.377(3)
C5–C6	1.413(8)	C9–C10	1.476(5)	C1–C2	1.392(2)	N1–C8	1.339(5)	C7–C9	1.442(3)
C9–C10	1.469(8)	C10–C11	1.324(5)	C4–C5	1.372(2)	C2–C3	1.381(8)	C9–C10	1.472(3)
C12–C17	1.401(9)	C11–C12	1.460(5)	C7–C9	1.457(2)	C6–C7	1.437(5)	C10–C11	1.328(3)
C16–C17	1.365(10)	C12–C13	1.385(5)	C12–C13	1.389(2)	C10–C11	1.307(5)	C11–C12	1.430(3)
N1–C8	1.349(7)	C12–C17	1.388(5)	C15–C16	1.379(2)	C13–C14	1.398(4)	C12–C13	1.344(3)
C2–C3	1.359(11)	C13–C14	1.372(5)	N1–C1	1.3869(19)	C15–C21	1.503(6)	C13–C14	1.404(3)
C6–C7	1.423(8)	C14–C15	1.372(6)	C1–C6	1.407(2)	N1–C18	1.478(5)	C14–C15	1.322(3)
C10–C11	1.320(9)	C15–C16	1.363(6)	C5–C6	1.399(2)	C3–C4	1.385(7)	C16–C17	1.485(3)
C13–C14	1.377(8)	C16–C17	1.382(6)	C9–C10	1.473(2)	C7–C8	1.372(5)	C15–O2–C12	106.08(18)

C18–C19	1.476(10)	C18–C19	1.491(6)	C12–C17	1.392(2)	C11–C12	1.472(4)	C8–N1–C1	108.26(16)
C1–N1–C8	108.9(5)	C8–N1–C1	108.5(3)	C16–C17	1.373(2)	C13–C20	1.518(4)	C8–N1–C16	128.54(19)
N1–C1–C2	129.1(6)	C8–N1–C18	127.0(3)	C15–O2–C18	117.63(14)	C16–C17	1.388(5)	C1–N1–C16	123.20(18)
C1–C2–C3	116.4(7)	C1–N1–C18	124.5(3)	C8–N1–C19	128.55(13)	C1–N1–C8	107.4(3)	N1–C1–C2	129.2(2)
C4–C5–C6	118.4(7)	C6–C1–N1	107.8(3)	C2–C1–C6	123.10(14)	N1–C1–C2	128.9(3)	N1–C1–C6	108.16(17)
C5–C6–C7	133.6(6)	C6–C1–C2	123.3(3)	C3–C4–C5	121.29(16)	C1–C2–C3	117.1(4)	C2–C1–C6	122.61(19)
C8–C7–C9	127.4(5)	N1–C1–C2	128.8(3)	C1–C6–C7	106.62(13)	C4–C5–C6	117.9(4)	C3–C2–C1	117.0(2)
O1–C9–C10	119.5(6)	C3–C2–C1	116.0(4)	C6–C7–C9	126.06(13)	C5–C6–C7	133.7(3)	C2–C3–C4	121.6(2)
C10–C11–C12	127.8(6)	C2–C3–C4	122.1(4)	O1–C9–C7	120.09(14)	C8–C7–C9	126.6(3)	C5–C4–C3	121.8(2)
C13–C12–C17	116.8(6)	C3–C4–C5	121.3(4)	C9–C10–C11	121.55(15)	O1–C9–C10	120.9(3)	C4–C5–C6	118.4(2)
Br1–C15–C14	119.2(5)	C4–C5–C6	118.5(4)	C11–C12–C17	119.20(14)	C10–C11–C12	128.9(3)	C5–C6–C1	118.57(19)
C15–C16–C17	120.7(6)	C1–C6–C5	118.8(3)	C13–C14–C15	119.38(15)	C13–C12–C17	118.6(3)	C5–C6–C7	134.5(2)
C1–N1–C18	122.9(5)	C1–C6–C7	107.0(3)	C14–C15–C16	119.61(15)	C14–C13–C20	118.6(3)	C1–C6–C7	106.91(17)
N1–C1–C6	107.4(5)	C5–C6–C7	134.2(3)	N1–C19–C20	113.36(13)	C14–C15–C21	120.7(3)	C8–C7–C9	127.37(19)
C2–C3–C4	122.2(7)	C8–C7–C6	105.5(3)	C1–N1–C8	108.51(12)	C12–C17–C16	118.9(3)	C8–C7–C6	105.39(17)
C1–C6–C5	117.9(6)	C8–C7–C9	128.4(3)	N1–C1–C2	129.03(14)	N1–C18–C19	113.1(3)	C9–C7–C6	127.23(18)
C6–C7–C8	104.9(5)	C6–C7–C9	125.9(3)	C1–C2–C3	116.38(16)	C1–N1–C18	127.5(3)	N1–C8–C7	111.27(18)
N1–C8–C7	110.5(5)	N1–C8–C7	111.2(3)	C4–C5–C6	118.86(16)	N1–C1–C6	108.6(3)	O1–C9–C7	120.85(19)
C7–C9–C10	119.3(5)	O1–C9–C7	120.1(3)	C5–C6–C7	135.10(15)	C2–C3–C4	121.6(4)	O1–C9–C10	119.95(19)
C11–C12–C13	122.8(6)	O1–C9–C10	120.6(3)	C8–C7–C9	127.91(14)	C1–C6–C5	119.4(3)	C7–C9–C10	119.20(18)
C12–C13–C14	122.3(6)	C7–C9–C10	119.3(3)	O1–C9–C10	120.94(15)	C6–C7–C8	104.9(3)	C11–C10–C9	121.8(2)
Br1–C15–C16	119.7(5)	C11–C10–C9	121.7(3)	C10–C11–C12	128.75(15)	N1–C8–C7	112.1(3)	C10–C11–C12	127.0(2)

C12–C17–C16	120.6(6)	C10–C11–C12	128.7(4)	C13–C12–C17	117.26(14)	C7–C9–C10	117.3(3)	C13–C12–O2	109.14(19)
C8–N1–C18	128.3(5)	C13–C12–C17	118.1(3)	O2–C15–C14	124.25(15)	C11–C12–C13	117.8(2)	C13–C12–C11	132.7(2)
C2–C1–C6	123.6(6)	C13–C12–C11	122.0(3)	C15–C16–C17	120.37(16)	C12–C13–C14	119.8(3)	O2–C12–C11	118.14(17)
C3–C4–C5	121.5(7)	C17–C12–C11	119.8(4)	C1–N1–C19	122.94(12)	C13–C14–C15	121.9(3)	C12–C13–C14	107.3(2)
C1–C6–C7	108.5(5)	C14–C13–C12	120.4(3)	N1–C1–C6	107.88(13)	C16–C15–C21	122.0(3)	C15–C14–C13	106.4(2)
C6–C7–C9	127.7(5)	C13–C14–C15	121.4(4)	C2–C3–C4	122.10(17)	C12–C17–C22	122.6(3)	C14–C15–O2	111.1(2)
O1–C9–C7	121.1(5)	C13–C14–Br1	119.1(3)	C1–C6–C5	118.28(14)	C8–N1–C18	125.1(3)	N1–C16–C17	113.9(2)
C9–C10–C11	122.6(6)	C15–C14–Br1	119.5(3)	C6–C7–C8	106.03(13)	C2–C1–C6	122.5(4)		
C11–C12–C17	120.4(6)	C16–C15–C14	118.6(4)	N1–C8–C7	110.96(13)	C3–C4–C5	121.5(5)		
C13–C14–C15	118.5(6)	C15–C16–C17	121.1(4)	C7–C9–C10	118.96(14)	C1–C6–C7	106.9(3)		
C14–C15–C16	121.2(6)	C16–C17–C12	120.4(4)	C11–C12–C13	123.54(14)	C6–C7–C9	128.3(3)		
N1–C18–C19	113.0(6)	N1–C18–C19	112.4(4)	C12–C13–C14	121.96(15)	O1–C9–C7	121.8(3)		
				O2–C15–C16	116.14(14)	C9–C10–C11	123.7(3)		
				C12–C17–	121.43(15)	C11–C12–C17	123.6(3)		

				C16					
						C12–C13–C20	121.7(2)		
						C14–C15–C16	117.3(3)		
						C15–C16–C17	123.6(3)		
						C16–C17–C22	118.5(3)		

Table S3. The calculated geometric parameters (bond distances and angles) of the studied compounds.

**3a**	**B3LYP**	**X-ray**	**3b**	**B3LYP**	**X-ray**	**3c**		
R(1–27)	1.916	1.904	R(1–25)	1.921	1.884	R(1–17)	1.229	1.23
R(2–17)	1.228	1.231	R(2–17)	1.228	1.229	R(2–27)	1.36	1.37
R(3–4)	1.393	1.375	R(3–4)	1.393	1.391	R(2–32)	1.422	1.417
R(3–15)	1.367	1.349	R(3–15)	1.366	1.344	R(3–4)	1.392	1.387
R(3–32)	1.464	1.473	R(3–32)	1.459	1.459	R(3–15)	1.368	1.35
R(4–5)	1.396	1.401	R(4–5)	1.396	1.394	R(3–36)	1.463	1.47
R(4–13)	1.415	1.384	R(4–13)	1.416	1.391	R(4–5)	1.396	1.392
R(5–6)	1.083	0.93	R(5–6)	1.084	0.931	R(4–13)	1.416	1.407
R(5–7)	1.389	1.359	R(5–7)	1.389	1.377	R(5–6)	1.084	0.93
R(7–8)	1.084	0.93	R(7–8)	1.084	0.93	R(5–7)	1.389	1.37

R(7–9)	1.406	1.376	R(7–9)	1.406	1.382	R(7–8)	1.084	0.93
R(9–10)	1.084	0.931	R(9–10)	1.084	0.93	R(7–9)	1.406	1.39
R(9–11)	1.388	1.382	R(9–11)	1.388	1.382	R(9–10)	1.084	0.93
R(11–12)	1.081	0.93	R(11–12)	1.081	0.93	R(9–11)	1.388	1.372
R(11–13)	1.402	1.413	R(11–13)	1.402	1.392	R(11–12)	1.081	0.93
R(13–14)	1.446	1.423	R(13–14)	1.447	1.445	R(11–13)	1.402	1.399
R(14–15)	1.385	1.383	R(14–15)	1.385	1.372	R(13–14)	1.446	1.439
R(14–17)	1.464	1.451	R(14–17)	1.464	1.446	R(14–15)	1.384	1.375
R(15–16)	1.076	0.931	R(15–16)	1.079	0.93	R(14–17)	1.467	1.457
R(17–18)	1.491	1.469	R(17–18)	1.492	1.476	R(15–16)	1.076	0.93
R(18–19)	1.084	0.931	R(18–19)	1.084	0.931	R(17–18)	1.487	1.473
R(18–20)	1.342	1.32	R(18–20)	1.342	1.323	R(18–19)	1.084	0.931
R(20–21)	1.087	0.931	R(20–21)	1.087	0.93	R(18–20)	1.344	1.329
R(20–22)	1.462	1.459	R(20–22)	1.463	1.46	R(20–21)	1.088	0.93
R(22–23)	1.406	1.4	R(22–23)	1.407	1.385	R(20–22)	1.459	1.458
R(22–30)	1.404	1.401	R(22–30)	1.404	1.388	R(22–23)	1.403	1.389
R(23–24)	1.083	0.93	R(23–24)	1.082	0.931	R(22–30)	1.409	1.392
R(23–25)	1.387	1.377	R(23–25)	1.385	1.371	R(23–24)	1.084	0.93
R(25–26)	1.082	0.93	R(25–26)	1.394	1.372	R(23–25)	1.39	1.381
R(25–27)	1.394	1.365	R(26–27)	1.082	0.931	R(25–26)	1.082	0.93
R(27–28)	1.39	1.363	R(26–28)	1.392	1.363	R(25–27)	1.401	1.386
R(28–29)	1.082	0.929	R(28–29)	1.084	0.931	R(27–28)	1.4	1.379
R(28–30)	1.391	1.364	R(28–30)	1.391	1.381	R(28–29)	1.083	0.93
R(30–31)	1.084	0.93	R(30–31)	1.084	0.931	R(28–30)	1.383	1.373
R(32–33)	1.094	0.97	R(32–33)	1.093	0.97	R(30–31)	1.085	0.93
R(32–34)	1.096	0.971	R(32–34)	1.092	0.97	R(32–33)	1.089	0.96
R(32–35)	1.523	1.476	R(32–35)	1.531	1.491	R(32–34)	1.095	0.96
R(35–36)	1.092	0.96	R(35–36)	1.093	0.96	R(32–35)	1.095	0.96
R(35–37)	1.092	0.96	R(35–37)	1.092	0.961	R(36–37)	1.095	0.969

R(35–38)	1.092	0.96	R(35–38)	1.092	0.96	R(36–38)	1.096	0.97
						R(36–39)	1.524	1.497
						R(39–40)	1.092	0.96
						R(39–41)	1.092	0.96
						R(39–42)	1.092	0.96
**R^2**	**0.991**			**0.99**			**0.996**	
A(1–27–25)	119.4	119.1	A(1–25–23)	119.2	119.1	A(1–17–14)	120.8	120.1
A(1–27–28)	119.6	119.7	A(1–25–26)	119	119.5	A(1–17–18)	121.2	120.9
A(2–17–14)	121.1	121.1	A(2–17–14)	121.2	120.1	A(27–2–32)	118.7	117.6
A(2–17–18)	120.8	119.5	A(2–17–18)	120.8	120.5	A(2–27–25)	124.7	124.2
A(4–3–15)	108.5	108.9	A(4–3–15)	108.6	108.4	A(2–27–28)	116	116.1
A(4–3–32)	123.6	122.9	A(4–3–32)	125.9	124.6	A(2–32–33)	105.8	109.5
A(3–4–5)	129.6	129.1	A(3–4–5)	129.9	128.8	A(2–32–34)	111.5	109.5
A(3–4–13)	108	107.3	A(3–4–13)	107.8	107.9	A(2–32–35)	111.5	109.5
A(15–3–32)	127.9	128.3	A(15–3–32)	125.5	127	A(4–3–15)	108.4	108.5
A(3–15–14)	110.7	110.5	A(3–15–14)	110.7	111.1	A(4–3–36)	123.7	122.9
A(3–15–16)	120.2	124.8	A(3–15–16)	119.4	124.4	A(3–4–5)	129.6	129
A(3–32–33)	107.6	108.9	A(3–32–33)	107.9	109.1	A(3–4–13)	108	107.9
A(3–32–34)	108.4	108.9	A(3–32–34)	107.3	109.2	A(15–3–36)	127.9	128.5
A(3–32–35)	114.1	113	A(3–32–35)	113.3	112.4	A(3–15–14)	110.7	111
A(5–4–13)	122.4	123.6	A(5–4–13)	122.3	123.3	A(3–15–16)	120.2	124.5
A(4–5–6)	121.9	121.8	A(4–5–6)	121.9	122	A(3–36–37)	107.7	108.9
A(4–5–7)	117.2	116.4	A(4–5–7)	117.3	116	A(3–36–38)	108.5	108.9
A(4–13–11)	119.2	117.9	A(4–13–11)	119.2	118.8	A(3–36–39)	114.1	113.4
A(4–13–14)	106.8	108.5	A(4–13–14)	106.9	107	A(5–4–13)	122.4	123.1
A(6–5–7)	120.9	121.7	A(6–5–7)	120.8	122	A(4–5–6)	121.9	121.8
A(5–7–8)	119.3	118.8	A(5–7–8)	119.3	119.1	A(4–5–7)	117.3	116.4

A(5–7–9)	121.2	122.2	A(5–7–9)	121.2	122	A(4–13–11)	119.2	118.3
A(8–7–9)	119.5	118.9	A(8–7–9)	119.5	118.9	A(4–13–14)	106.8	106.6
A(7–9–10)	119.1	119.3	A(7–9–10)	119.1	119.3	A(6–5–7)	120.9	121.8
A(7–9–11)	121.4	121.4	A(7–9–11)	121.3	121.3	A(5–7–8)	119.3	119
A(10–9–11)	119.5	119.3	A(10–9–11)	119.5	119.4	A(5–7–9)	121.2	122.1
A(9–11–12)	121.9	120.8	A(9–11–12)	121.9	120.7	A(8–7–9)	119.5	118.9
A(9–11–13)	118.6	118.4	A(9–11–13)	118.6	118.5	A(7–9–10)	119.1	119.4
A(12–11–13)	119.5	120.8	A(12–11–13)	119.5	120.8	A(7–9–11)	121.4	121.3
A(11–13–14)	134	133.6	A(11–13–14)	133.9	134.2	A(10–9–11)	119.5	119.3
A(13–14–15)	106.1	104.9	A(13–14–15)	106	105.5	A(9–11–12)	122	120.6
A(13–14–17)	126	127.7	A(13–14–17)	126	125.8	A(9–11–13)	118.6	118.9
A(15–14–17)	127.9	127.4	A(15–14–17)	128	128.4	A(12–11–13)	119.4	120.6
A(14–15–16)	129.2	124.7	A(14–15–16)	129.9	124.4	A(11–13–14)	134	135.1
A(14–17–18)	118.1	119.3	A(14–17–18)	118	119.3	A(13–14–15)	106.1	106
A(17–18–19)	118.5	118.8	A(17–18–19)	118.5	119.1	A(13–14–17)	126	126.1
A(17–18–20)	120.3	122.5	A(17–18–20)	120.2	121.7	A(15–14–17)	127.9	127.9
A(19–18–20)	121.2	118.7	A(19–18–20)	121.3	119.2	A(14–15–16)	129.1	124.5
A(18–20–21)	115.7	116.1	A(18–20–21)	115.8	115.7	A(14–17–18)	118.1	119
A(18–20–22)	128.2	127.8	A(18–20–22)	128.2	128.7	A(17–18–19)	118.5	119.2
A(21–20–22)	116.1	116.1	A(21–20–22)	116	115.6	A(17–18–20)	120.4	121.5
A(20–22–23)	123.6	122.8	A(20–22–23)	122.9	122.1	A(19–18–20)	121.1	119.3
A(20–22–30)	118.7	120.4	A(20–22–30)	118.8	119.8	A(18–20–21)	115.5	115.6
A(23–22–30)	117.7	116.8	A(23–22–30)	118.3	118.1	A(18–20–22)	128.5	128.8
A(22–23–24)	120.1	118.8	A(22–23–24)	120.6	119.8	A(21–20–22)	116	115.6
A(22–23–25)	121.4	122.2	A(22–23–25)	119.9	120.4	A(20–22–23)	123.8	123.5
A(22–30–28)	121.7	120.6	A(22–30–28)	121	120.4	A(20–22–30)	119	119.2
A(22–30–31)	119.1	119.7	A(22–30–31)	119	119.8	A(23–22–30)	117.2	117.3
A(24–23–25)	118.6	118.9	A(24–23–25)	119.6	119.8	A(22–23–24)	119.8	119
A(23–25–26)	120.5	120.8	A(23–25–26)	121.8	121.4	A(22–23–25)	121.8	122

A(23–25–27)	119.3	118.5	A(25–26–27)	120.5	120.7	A(22–30–28)	121.8	121.4
A(26–25–27)	120.2	120.7	A(25–26–28)	118.4	118.6	A(22–30–31)	118.9	119.3
A(25–27–28)	121	121.2	A(27–26–28)	121	120.7	A(24–23–25)	118.4	119
A(27–28–29)	120.5	119.7	A(26–28–29)	119.4	119.5	A(23–25–26)	119.3	120.3
A(27–28–30)	119	120.7	A(26–28–30)	120.5	121	A(23–25–27)	119.8	119.4
A(29–28–30)	120.6	119.6	A(29–28–30)	120	119.5	A(26–25–27)	120.9	120.3
A(28–30–31)	119.2	119.7	A(28–30–31)	119.9	119.8	A(25–27–28)	119.4	119.6
A(33–32–34)	107	107.7	A(33–32–34)	106.8	107.8	A(27–28–29)	118.5	119.8
A(33–32–35)	109.6	109	A(33–32–35)	110.7	109.1	A(27–28–30)	120	120.4
A(34–32–35)	109.9	109	A(34–32–35)	110.5	109.1	A(29–28–30)	121.5	119.8
A(32–35–36)	108.9	109.4	A(32–35–36)	110	109.5	A(28–30–31)	119.3	119.3
A(32–35–37)	112	109.5	A(32–35–37)	110.8	109.4	A(33–32–34)	109.3	109.5
A(32–35–38)	111.6	109.6	A(32–35–38)	111.2	109.5	A(33–32–35)	109.3	109.5
A(36–35–37)	107.5	109.4	A(36–35–37)	108.3	109.5	A(34–32–35)	109.5	109.5
A(36–35–38)	107.8	109.5	A(36–35–38)	108.3	109.5	A(37–36–38)	107	107.7
A(37–35–38)	108.8	109.4	A(37–35–38)	108.1	109.5	A(37–36–39)	109.5	108.9
						A(38–36–39)	109.8	108.9
						A(36–39–40)	108.9	109.5
						A(36–39–41)	111.6	109.4
						A(36–39–42)	111.9	109.5
						A(40–39–41)	107.9	109.4
						A(40–39–42)	107.6	109.5
						A(41–39–42)	108.7	109.5
**R^2**	**0.978**			**0.979**			**0.982**	
**3d**	**B3LYP**	**X-ray**	**3e**	**B3LYP**	**X-ray**			
R(1–16)	1.228	1.227	R(1–17)	1.229	1.235			
R(2–3)	1.392	1.384	R(2–22)	1.374	1.367			

R(2–14)	1.367	1.339	R(2–27)	1.358	1.358
R(2–29)	1.459	1.478	R(3–4)	1.392	1.386
R(3–4)	1.397	1.388	R(3–15)	1.367	1.353
R(3–12)	1.416	1.39	R(3–29)	1.463	1.46
R(4–5)	1.084	0.93	R(4–5)	1.396	1.392
R(4–6)	1.389	1.381	R(4–13)	1.415	1.4
R(6–7)	1.084	0.93	R(5–6)	1.084	0.931
R(6–8)	1.406	1.385	R(5–7)	1.389	1.367
R(8–9)	1.084	0.93	R(7–8)	1.084	0.929
R(8–10)	1.388	1.38	R(7–9)	1.406	1.379
R(10–11)	1.081	0.93	R(9–10)	1.084	0.931
R(10–12)	1.402	1.41	R(9–11)	1.388	1.376
R(12–13)	1.447	1.437	R(11–12)	1.081	0.929
R(13–14)	1.384	1.372	R(11–13)	1.402	1.399
R(13–16)	1.467	1.442	R(13–14)	1.445	1.447
R(14–15)	1.079	0.93	R(14–15)	1.385	1.377
R(16–17)	1.49	1.486	R(14–17)	1.465	1.442
R(17–18)	1.081	0.93	R(15–16)	1.076	0.93
R(17–19)	1.344	1.307	R(17–18)	1.486	1.472
R(19–20)	1.087	0.93	R(18–19)	1.083	0.93
R(19–21)	1.468	1.472	R(18–20)	1.345	1.327
R(21–22)	1.42	1.395	R(20–21)	1.086	0.93
R(21–28)	1.416	1.415	R(20–22)	1.433	1.429
R(22–23)	1.391	1.398	R(22–23)	1.373	1.344
R(22–36)	1.512	1.518	R(23–24)	1.078	0.93
R(23–24)	1.086	0.931	R(23–25)	1.425	1.404
R(23–25)	1.396	1.39	R(25–26)	1.078	0.93
R(25–26)	1.392	1.361	R(25–27)	1.361	1.321
R(25–40)	1.509	1.503	R(27–28)	1.077	0.929

R(26–27)	1.086	0.93	R(29–30)	1.095	0.97
R(26–28)	1.397	1.388	R(29–31)	1.095	0.97
R(28–44)	1.512	1.51	R(29–32)	1.523	1.485
R(29–30)	1.092	0.969	R(32–33)	1.092	0.961
R(29–31)	1.093	0.971	R(32–34)	1.092	0.96
R(29–32)	1.531	1.469	R(32–35)	1.092	0.959
R(32–33)	1.093	0.96			
R(32–34)	1.092	0.96			
R(32–35)	1.092	0.96			
R(36–37)	1.091	0.96			
R(36–38)	1.095	0.96			
R(36–39)	1.093	0.959			
R(40–41)	1.096	0.96			
R(40–42)	1.092	0.96			
R(40–43)	1.093	0.96			
R(44–45)	1.091	0.96			
R(44–46)	1.092	0.961			
R(44–47)	1.095	0.96			
**R^2**	**0.995**			**0.995**	
A(1–16–13)	120.9	121.8	A(1–17–14)	121.1	120.8
A(1–16–17)	121.2	120.9	A(1–17–18)	120.9	120
A(3–2–14)	108.6	107.4	A(22–2–27)	107.3	106.1
A(3–2–29)	125.9	127.5	A(2–22–20)	119.2	118.1
A(2–3–4)	129.9	128.9	A(2–22–23)	109.1	109.1
A(2–3–12)	107.8	108.6	A(2–27–25)	110.7	111.1
A(14–2–29)	125.5	125.1	A(2–27–28)	115.9	124.4
A(2–14–13)	110.7	112.1	A(4–3–15)	108.5	108.3

A(2–14–15)	119.4	123.9	A(4–3–29)	123.6	123.2
A(2–29–30)	107.4	109	A(3–4–5)	129.6	129.3
A(2–29–31)	108	108.9	A(3–4–13)	108	108.2
A(2–29–32)	113.4	113.1	A(15–3–29)	127.9	128.5
A(4–3–12)	122.3	122.5	A(3–15–14)	110.6	111.3
A(3–4–5)	121.9	121.4	A(3–15–16)	120.3	124.4
A(3–4–6)	117.3	117.1	A(3–29–30)	108.1	108.8
A(3–12–10)	119.2	119.4	A(3–29–31)	107.9	108.8
A(3–12–13)	106.9	106.9	A(3–29–32)	114.1	113.9
A(5–4–6)	120.8	121.5	A(5–4–13)	122.4	122.6
A(4–6–7)	119.3	119.2	A(4–5–6)	121.9	121.5
A(4–6–8)	121.2	121.6	A(4–5–7)	117.3	117.1
A(7–6–8)	119.5	119.2	A(4–13–11)	119.2	118.6
A(6–8–9)	119.1	119.3	A(4–13–14)	106.8	106.9
A(6–8–10)	121.3	121.5	A(6–5–7)	120.8	121.5
A(9–8–10)	119.5	119.2	A(5–7–8)	119.3	119.2
A(8–10–11)	121.9	121.1	A(5–7–9)	121.2	121.6
A(8–10–12)	118.6	117.9	A(8–7–9)	119.5	119.2
A(11–10–12)	119.5	121	A(7–9–10)	119.1	119.1
A(10–12–13)	133.9	133.7	A(7–9–11)	121.4	121.8
A(12–13–14)	106	104.9	A(10–9–11)	119.5	119.1
A(12–13–16)	126	128.3	A(9–11–12)	121.9	120.8
A(14–13–16)	128	126.6	A(9–11–13)	118.6	118.4
A(13–14–15)	129.8	123.9	A(12–11–13)	119.5	120.8
A(13–16–17)	117.9	117.3	A(11–13–14)	134	134.5
A(16–17–18)	118.4	118	A(13–14–15)	106.1	105.4
A(16–17–19)	120.2	123.7	A(13–14–17)	126.1	127.2
A(18–17–19)	121.4	118.3	A(15–14–17)	127.8	127.4
A(17–19–20)	114.3	115.6	A(14–15–16)	129.1	124.4

A(17–19–21)	129.2	128.9	A(14–17–18)	118	119.2
A(20–19–21)	116.4	115.5	A(17–18–19)	119.8	119.1
A(19–21–22)	117.5	117.7	A(17–18–20)	120.2	121.8
A(19–21–28)	123.6	123.6	A(19–18–20)	120	119.1
A(22–21–28)	118.9	118.6	A(18–20–21)	117.5	116.5
A(21–22–23)	119.7	119.8	A(18–20–22)	126.4	127
A(21–22–36)	121.3	121.7	A(21–20–22)	116.1	116.5
A(21–28–26)	119.1	118.9	A(20–22–23)	131.7	132.7
A(21–28–44)	123.2	122.6	A(22–23–24)	125.6	126.4
A(23–22–36)	118.9	118.5	A(22–23–25)	106.9	107.3
A(22–23–24)	118.8	119.1	A(24–23–25)	127.5	126.4
A(22–23–25)	121.9	121.9	A(23–25–26)	127.6	126.8
A(22–36–37)	110.5	109.5	A(23–25–27)	106	106.4
A(22–36–38)	111.7	109.5	A(26–25–27)	126.4	126.8
A(22–36–39)	111.9	109.4	A(25–27–28)	133.4	124.5
A(24–23–25)	119.2	119	A(30–29–31)	107	107.6
A(23–25–26)	117.8	117.3	A(30–29–32)	109.8	108.8
A(23–25–40)	120.9	120.7	A(31–29–32)	109.7	108.8
A(26–25–40)	121.3	122	A(29–32–33)	108.9	109.4
A(25–26–27)	119	118.2	A(29–32–34)	111.7	109.5
A(25–26–28)	122.5	123.6	A(29–32–35)	111.8	109.5
A(25–40–41)	111	109.4	A(33–32–34)	107.8	109.5
A(25–40–42)	111.4	109.5	A(33–32–35)	107.7	109.5
A(25–40–43)	111.4	109.5	A(34–32–35)	108.7	109.5
A(27–26–28)	118.5	118.2			
A(26–28–44)	117.7	118.5			
A(28–44–45)	112	109.5			
A(28–44–46)	110.2	109.5			

A(28–44–47)	111.8	109.5	
A(30–29–31)	106.8	107.8	
A(30–29–32)	110.5	108.9	
A(31–29–32)	110.6	108.9	
A(29–32–33)	110.1	109.4	
A(29–32–34)	110.8	109.4	
A(29–32–35)	111.2	109.4	
A(33–32–34)	108.3	109.5	
A(33–32–35)	108.3	109.5	
A(34–32–35)	108.1	109.6	
A(37–36–38)	107.6	109.5	
A(37–36–39)	108	109.4	
A(38–36–39)	106.9	109.5	
A(41–40–42)	107.5	109.4	
A(41–40–43)	107.2	109.5	
A(42–40–43)	108.1	109.5	
A(45–44–46)	108.4	109.4	
A(45–44–47)	107.3	109.5	
A(46–44–47)	107	109.4	
	**0.974**		**0.968**

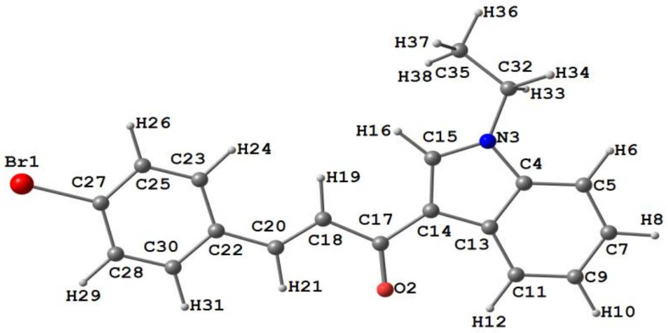

3a

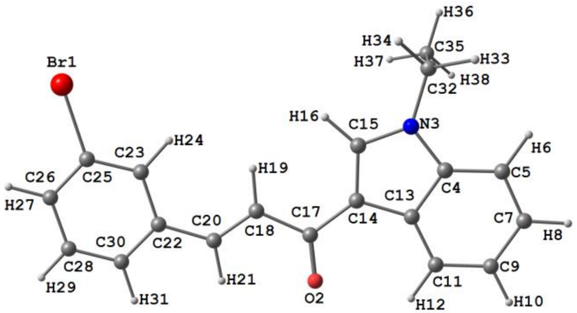

3b

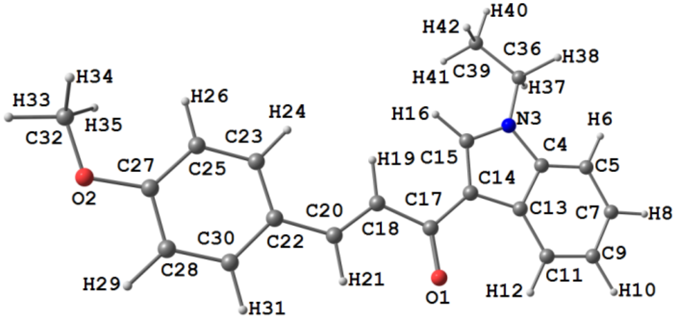

3c

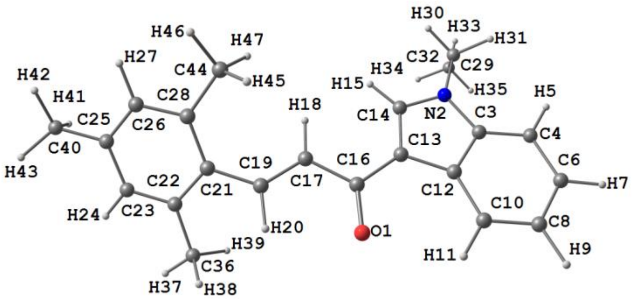

3d

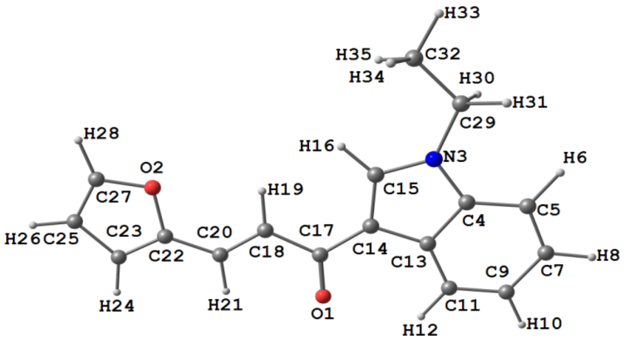

3e

Figure S1. The optimized molecular structures of the studied molecules.
